# Temporary business model innovation – SMEs’ innovation response to the Covid‐19 crisis

**DOI:** 10.1111/radm.12498

**Published:** 2021-08-26

**Authors:** Thomas Clauss, Matthias Breier, Sascha Kraus, Susanne Durst, Raj V. Mahto

**Affiliations:** ^1^ Department of Management and Entrepreneurship Witten/Herdecke University Alfred‐Herrhausen‐Str. 50 Witten 58455 Germany; ^2^ Department of Technology and Innovation University of Southern Denmark Odense Denmark; ^3^ Lappeenranta University of Technology Lappeenranta 53850 Finland; ^4^ Faculty of Economics & Management Free University of Bozen‐Bolzano Bolzano 39100 Italy; ^5^ Department of Business Administration Tallinn University of Technology Tallinn 12616 Estonia; ^6^ Anderson School of Management The University of New Mexico Albuquerque New Mexico 87131 USA

## Abstract

The Covid‐19 crisis has hit SMEs particularly hard. Numerous business models (BM) have been limited or rendered downright impossible due to decreased social contact. SMEs can respond to this exogenous crisis via temporary business model innovation (BMI). This empirical study investigates these temporary BMs using a multiple case study approach based on five SMEs in Austria, Germany, and Liechtenstein who within a short period of time applied their core competencies and networks to integrate new BMs, which were in some cases very different from existing ones. These had a positive effect on strategic flexibility, and if desired can also be incorporated into the firm long‐term. The paper contributes to SME crisis management during the Covid‐19 pandemic by pointing out and developing a successful management mechanism that allows to survive a crisis or even improve during this time. Moreover, we contribute to BMI literature by explaining temporary BMI as a new form of BMI. It also makes clear to managers that temporary BMs add value to firms and create new revenue streams.

## 1 Introduction

The Covid‐19 pandemic has disrupted social life and economic activity across the globe (Clark et al., 2020). The economic impact has been unprecedented, with most countries experiencing large‐scale job losses and economic contraction (e.g., in Q1 of 2020, China’s GDP shrunk by 6.8%, while the euro area saw GDP drop by 3.8% [Chen et al., 2020]). In comparison to previous crises, the Covid‐19 pandemic has caused a simultaneous demand and supply shock (del Rio‐Chanona et al., 2020). For many firms, lockdowns and strict regulations have challenged existing business models (BMs) (Ritter and Pedersen, 2020; Breier et al., 2021), while some firms have not been able to pursue their established business operations at all. This situation has called for a drastic, rapid crisis management response.

Small and medium‐sized firms (SMEs) are particularly vulnerable to crises (Shepherd, 2003; Kraus et al., 2013). Resource scarcity and lack of preparedness have restricted their strategic choices in managing the Covid‐19 crisis (Eggers, 2020). SMEs are usually not diversified, but instead rely on only one specific BM (Pal et al., 2012). Furthermore, banks associate SMEs with higher risks, thus limiting their options for debt financing during crises (Piette and Zachary, 2015). On the other hand, SMEs are flexible, entrepreneurial, and embedded in communities, and have been shown to possess unique capabilities to mount an effective response to a crisis and ultimately emerge stronger (Ter Wengel and Rodriguez, 2006; Dahles and Susilowati, 2015). Crisis management studies have primarily explored which characteristics and strategies have helped SMEs survive (Eggers, 2020). Factors such as young firm age (Simón‐Moya et al., 2016), management expertise (Giannacourou et al., 2015), and market orientation (Petzold et al., 2019) have been shown to be positively related to SMEs’ crisis performance. Studies have also concluded that SMEs’ innovative stances and entrepreneurial orientation are helpful for surviving a crisis (e.g., Eggers and Kraus, 2011; Vargo and Seville, 2011). Beliaeva et al. (2020) relate this effect to their ability to identify opportunities during crises. But despite these initial findings, surprisingly little is known about how SMEs can best cope with severe crises.

We add to this research discourse by analyzing the reactions of SMEs to the Covid‐19 crisis. This pandemic provides a unique context for studying how firms can cope with a crisis if their existing BM suddenly becomes infeasible. Wenzel et al. (2020) proposed four different strategies for firms to respond to a crisis: retrenchment, persevering, innovating, and exit. In line with the arguments above, we focus our study on the opportunity for SMEs to overcome a crisis through innovation and temporary business model innovation (BMI). We argue that as the unique characteristics of this crisis have seriously affected the BMs of many SMEs, these firms have had to come up with new BMs at least for the duration of the crisis. In contrast to previous research that suggests that dual or multiple BMs can help firm diversification (e.g., Markides and Charitou, 2004; Aversa et al., 2017; Winterhalter et al., 2016), this context has created a situation in which a new BM is temporarily required to survive the crisis while the established BM is significantly reduced or even placed on hold. Against this background, the research objective of this study is to explore how SMEs pursue temporary BMI in response to Covid‐19 and what effects this has. This question aims to achieve a deeper understanding of SMEs’ crisis management mechanisms.

By analyzing the case data of five firms who had a temporary BMI early in the Covid‐19 crisis, we make two important contributions to the literature. First, we add to the SME crisis management literature (e.g., Eggers, 2020) and the growing stream of studies proposing innovation and temporary BMI as potential strategies to cope with the Covid‐19 crisis (Chesbrough, 2020; Kraus et al., 2020a; Wenzel et al., 2020). Especially, SMEs due to their liabilities (Eggers, 2020) have to engage in innovative strategies to survive in the long run. Second, we contribute to the literature on BMI by showing that temporary BMI which relies on existing core competencies and is positioned in nascent industries (Zook and Allen, 2003) is a viable way to change an organization as a response to changing external conditions. In contrast to existing research (e.g., Clauss et al., 2021), we demonstrate that BMI as a response to changing external circumstances must not necessarily be radical and irreversible but can be a more tactical approach to temporary adapting the organization e.g., if a crisis is to be faced.

## 2 Theoretical foundation

### 2.1 Firms’ response to a crisis

According to Pearson and Clair (1998), an organizational crisis is defined as ‘…a low probability, high‐impact event that threatens the viability of the organization’ (p. 60). The Institute for Crisis Management (ICM) (2004) divides crises into two primary types: sudden and smoldering. Sudden crises are the unexpected external events in which the organization has virtually no control and limited fault or responsibility. The Covid‐19 pandemic can be viewed as an example of this type of crisis. Smoldering crises are those events that start out as small, internal problems within a firm, become public at some point, and over time escalate as a result of inattention and/or poor decisions by management.

Although no two crises are alike, research suggests that they all have three common elements: surprise, threat, and short response time (Williams et al., 2017). Scholars argue (e.g., Smith and Riley, 2012) that regardless of the type of crisis, it requires immediate and decisive action by an organization. Hence, crisis management has been defined as ‘the systematic way in which members of an organization, in conjunction with external stakeholders, work to avoid potential crises and to minimize and resolve those that do occur’ (Brumfield, 2012, p. 45). The available normative guidance on managing a crisis suggests that managers should be prepared to make available more time and resources to expand the required operating space (Bowers et al., 2017). The need for continued internal and external communication has been frequently highlighted here as well (Gilstrap et al., 2016; Bowers et al., 2017).

Wenzel et al. (2020) proposed four strategic responses to a crisis: retrenchment, persevering, innovating, and exit. These strategies were shown as effectively capturing the early responses of family firms to the Covid‐19 pandemic (Kraus et al., 2020a). Retrenchment involves cost‐cutting measures that may reduce the scope of a firms’ business activities. This strategy appears to support firms in surviving a crisis in the short run. Persevering is about preserving the status quo of a firms’ business activities. This may be achieved through debt financing, and seems suitable in response to a crisis in the medium run, even though it may threaten the long‐term survival of the firm. Exit means the discontinuation of a firm’s business activities; it is a strategy which is not limited to a crisis, and can be selected at any time. Finally, innovating means that the firm engages in strategic renewal in response to a crisis.

In contrast to the other mechanisms, an innovation strategy to crisis management is the most future‐oriented because it may provide solutions on how to use opportunities that emerge from a crisis. It has been shown that firms pursuing more explorative strategies toward new product and market developments are those that cope better with crises (Archibugi et al., 2013). In line with this, studies have shown that SMEs’ innovative stances and entrepreneurial orientations are helpful for surviving a crisis (e.g., Eggers and Kraus, 2011; Vargo and Seville, 2011). Considering how the Covid‐19 crisis created a situation in which the existing BMs of many firms were suddenly placed on hold, managers should assess the impact of the crisis on their firms’ BM (Ritter and Pedersen, 2020) and may potentially seek out BMI as an effective opportunity (Kraus et al., 2020a).

### 2.2 Temporary BMI

The BM concept has received considerable theoretical (Massa et al., 2017) and practical (Pohle and Chapman, 2006) interest because it provides a useful perspective for understanding a firm’s business and competitive logic. Scholars recently agreed that BMs are conceptualized as configurations of the three interrelated key elements of value proposition, value creation, and value capture (Clauss, 2017; Foss and Saebi, 2017). These elements are configured as mutually enforcing systems that together define the gestalt of the organization (Martins et al., 2015; Kulins et al., 2016). BMI is then defined as ‘designed, nontrivial changes to the key elements of a firm’s BM and/or the architecture linking these elements’ (Foss and Saebi, 2017, p. 207). BMI extends the scope of product and process innovation as key elements of firms’ organization, along with when their configurations are changed (Foss and Saebi, 2017; Clauss et al., 2020). As a result, it provides firms with the opportunity to create novel activities that go beyond product and process innovation (Osiyevskyy and Dewald, 2015). Studies show that competitive advantages can be achieved either by innovating components of the BM or the entire BM (Berends et al., 2016; Clauss et al., 2020). BMI therefore not only exists if radical changes are implemented, but can also be the result of their more incremental reconfigurations (Velu and Jacob, 2016; Foss and Saebi, 2017; Kraus et al., 2020b). Foss and Saebi (2017) differentiate BMI in terms of their newness and scope.^1^ The first dimension captures the ‘degree of novelty of the BMI.’ It differentiates whether a BMI is only new for the firm (Johnson et al., 2008; Bock et al., 2012), or if it is completely new to the whole industry (Santos et al., 2009). The second dimension captures the BMI’s scope, defining how much of the existing BM is affected by the innovation. In line with the above‐mentioned ideas, the scope is the number of BM elements (i.e., value creation, value proposition, and value capture) that are changed by the BMI. If only one or a few elements of the BM are changed, the scope of the BMI would be modular, whereas the orchestrated reconfiguration of all elements of the BM would be termed *architectural BMI* (Foss and Saebi, 2017). The more components that are changed and the greater the novelty of the changes outside of the firm, the more radical the BMI is. On the other hand, incremental BMIs are based on only modular improvements of a firm’s existing BM.

Because complex BMIs require fundamental changes in the organizational system (Berends et al., 2016), involve substantial redeployment of resources (Doz and Kosonen, 2010), and are usually the consequence of a long‐term strategy defined by the firm (Casadesus‐Masanell and Ricart, 2010), previous literature predominantly considered BMI as a firm’s enduring reconfiguration. Paradoxically, another stream of literature suggests that BMI may be achieved by temporarily experimenting with new BMs (Sosna et al., 2010; Andries et al., 2013) or by creating temporary spin‐off BMs (Chesbrough and Rosenbloom, 2002; Clausen and Rasmussen, 2013; Markides, 2013) that may be reintegrated into the parent firm at a later date. Furthermore, it has often been proposed in start‐ups that new BMs may require regular pivots in which their elements are adjusted based on market feedback (Felin et al., 2019). With these studies in mind, we propose that BMI does not inevitably lead to an enduring reconfiguration of the organization, but that temporary BMIs are often possible or necessary for a period of time. We regard a *temporary business model innovation* as one which is, at least at the time of its origination, not intended as permanent. These temporary BMIs are assumed to be particularly appropriate if firms’ operating conditions significantly change, and more modular and less novel changes in the elements of the BM can recreate or improve the competitive position.

In contrast to more radical BMI, temporary BMI should be closely related to the strategy and core competencies (Prahalad and Hamel, 1990) of the firm. In line with Zook and Allen (2003), BMIs take place in adjacent spaces where firms can reuse existing capabilities. Casadesus‐Masanell (2010, 2010) shows how BMs form as a consequence of strategic decisions, and as rather rigid configurations of the organizational activity system. These structures however leave some residual freedom for changes as a direct response to changing external conditions, and as long as these changes are in line with the core competencies and strategic orientation of the firm. Zott and Amit (2008) in a similar vein show that the alignment of the BM and the established product‐market strategy is important for sustained competitive performance. In turn, if BMIs are consistent with the firm’s core competencies, their limited strategic complexity may make these more reversible, and they may be partially or fully undone if the external conditions return to the previous state. Whatever the case, temporary BMIs can still represent an optimal‐use case or prototype for long‐term BM changes.

Temporary BMI may not necessarily substitute for the existing BM. In situations where the established BM cannot be pursued due to external conditions (e.g., regulations), new BMs will exist in parallel with the established BM. Previous research has emphasized that tensions between two parallel BMs may arise when they follow competing institutional logics (e.g., low cost vs premium) (Winterhalter et al., 2016) or if they are competing for limited resources (Markides and Charitou, 2004). Studies have shown that to overcome these potential issues, new (temporary) parallel BMs should be able to share resources and activities (Snihur and Tarzijan, 2018), and should furthermore be compatible and synergistic regarding value chain linkages and technologies (Aversa et al., 2017).

We propose that temporary BMI are a potential mechanism for firms in responding to dramatic changes (i.e., demand and supply shocks). If they are the result of the Covid‐19 pandemic, they are a kind of ‘extended environment.’ Initial evidence by Kraus et al. (2020a) indicate that European family firms utilized temporary changes in their BM as a direct response to the Covid‐19 crisis. Furthermore, Ritter and Pedersen (2020) have recommended that in response to the crisis, firms should consider changes in a few core dimensions of the BMs. Gutierrez‐Gutierrez et al. (2020) called for rapid responsible innovation as a sustainable response strategy to the Covid‐19 pandemic. Following these initial ideas, together with the theoretical background of temporary BMI, we examined five firms that engaged in temporary BMI as a direct response to the Covid‐19 crisis.

## 3 Methodology

This study explores temporary BMIs in SMEs during crises. Kraus et al. (2020a) researched how firms adapt their BMs as a short‐term response to cope with crises. Although they reflected their findings into extant literature, that of Casadeus and Ricart (2010), the understanding of temporary BMIs in SMEs is underdeveloped. This argued in favor of an exploratory multiple case study approach. A case study ‘attempts to examine: (a) a contemporary phenomenon in its real‐life context, especially when (b) the boundaries between phenomenon and context are not clearly evident’ (Yin, 1981, p. 59). This approach can be used to find answers to ‘how’ or ‘why’ questions and addresses real‐life problems (Yin, 2009). Furthermore, a case study design enables the investigation of complex relationships and provides a basis for the development of theories (Flyvbjerg, 2006). The use of multiple cases was considered effective for theory development, and the underlying replication logic increases the likelihood of producing more robust and generalizable theory (Eisenhardt and Graebner, 2007).

### 3.1 Selection of cases

The sample in this study included SMEs from Austria, Germany, and Liechtenstein. The search for and selection of suitable SMEs was based on theoretical sampling (Eisenhardt and Graebner, 2007). We identified companies that were negatively affected by the crisis and were thus in a need to strategically deal with the situation. Therefore, all our cases come from low‐tech industries which were more severely affected by the crisis. Here we identified SMEs that responded to the crisis via an innovation strategy (Wenzel et al., 2020) and operated with a temporary BMI (Kraus et al., 2020a). With help from the local Chambers of Commerce, we identified firms who at the time of the study were already known to have innovated their existing BMs as a response to Covid‐19. A total of twelve possible firms were identified based on this. After double‐checking whether these firms were really engaged in temporary BMIs, all of them were approached and asked whether they would participate in our study, with five of them agreeing to do so.

### 3.2 Data collection and analysis

The data for the present study were collected from various sources, i.e., through semi‐structured interviews; follow up calls; archive data such as e‐mails, internal reports, and presentations; and the firms’ websites and social media activity on Instagram and Facebook as well as on‐sight visits and observation of the new BMs. In doing this, we used multiple sources of evidence as recommended by Yin (2009) to increase the overall quality of the case study and triangulated the results of the interviews wherever possible.

An interview guide was created for the semi‐structured interviews. Accordingly, a number of focal topics were specified at the outset of the interviews. Very specific questions had to be asked to develop the necessary in‐depth information about the new BMs, their basis and initial effects, and to explore the underdeveloped BMI phenomenon (Guest et al., 2006). More precisely, the content of the interview guide focused on how the participating SMEs had dealt with the crisis to date; which measures were chosen and why; what the temporary BM looked like; how the process of implementation worked; and whether the firms had already been exposed to significant consequences and changes resulting from their temporary BMI. Given the exploratory character of the present study, it was not possible to rely on existing questionnaires, requiring new questions to be formulated. The use of semi‐structured interviews allowed the analysis of a complex situation and the inclusion of the experiences and views of the participants involved in it (Graebner et al., 2012). A total of eight interviews were conducted between April 30th and May 6th of 2020. This was during a time when the countries in the study had just started to slowly reopen following their Covid‐19 lockdowns. Due to the still‐active social distancing measures and restricted border access, the interviews were conducted via the digital tool LoopUp, recorded with the consent of the participants, and later transcribed. The interviewees also enabled access to further material including firm reports, sales lists, and marketing material. The interviewees furthermore agreed to be contacted at a later date for any follow‐up questions.

The data analysis primarily followed inductive reasoning (Creswell and Poth, 2016). It also took advantage of the underlying ideas of thematic analysis. This approach to data analysis searches for topics that appear important for the understanding of the phenomenon in focus (Fereday and Muir‐Cochrane, 2006). Data reduction is here supported through segmenting, categorizing, and summarizing relevant concepts within the data set being examined (Ayres, 2008).

The data analysis began by a research team member transcribing the recorded interviews. This researcher took notes during this process, which supported not only the recording of why certain data chunks were assigned to particular topics, but the initial data interpretation as well. Once all transcripts were generated, the first step was to identify all data related to a list of predetermined topics covering three areas: (1) variance between the existing and the temporary BM, (2) changes in BM elements, and (3) time perspective of the BM. Additional codes were assigned to the portions of data that represented new topics (Saunders, 2012), further underlining the selected inductive approach. This was done for each case. New topics emerged as a result: branding and reputation issues, use of core competencies, effects on the existing BMs, and effects on the strategic flexibility of a firm. To reduce the danger of misinterpretation, all of the authors read through the transcripts and discussed the findings. Even though data from other sources was gathered and additionally included in the above‐mentioned process, priority was given to the semi‐structured interviews. Once this process was completed, individual case reports were prepared for each firm involved, which also formed the basis for a within‐case analysis to be followed (Eisenhardt, 1989). The cross‐case analysis aimed at highlighting the differences and similarities between the cases involved (Eisenhardt and Graebner, 2007), which was supported by the topics identified in the previous step. Moreover, comparison tables were produced to enable the cross‐case analysis. This also helped the researchers discuss the findings and agree on the relevant topics needed to address the research aim. The detailed case descriptions can be found in the Supplementary Information.

## 4 Findings

### 4.1 Within‐case analysis

In the following section we are providing an overview of the main BM changes that were conducted by each of our case companies. Table 1 provides a comprehensive overview on the changes in value proposition, value creation and value capture of the old and the new BM. Each case description highlights the new BM, the evaluation of the BMI based on Foss and Saebi (2017), and some general data about the case.

**Table 1:** Description of old and new BM based on the three BMI dimensions

Case		Value proposition	Value creation	Value capture
Case A	Old	Production of a consumer good	Production of alcoholic beverages from basic ingredients including bottling for customers	Sell the produced spirits primarily to businesses and partly to retail customers via an online shop
	New	Produce disinfectant	Production of disinfectant to tackle health crisis and fill the increased demand during the crisis	Sell the disinfectant to businesses
Case B	Old	Network people in a specialized business environment.	Host local events and network the sponsors with the people they want to meet	Sponsors pay to be invited to the dinners that take place around the event
	New	Network people in a specialized business environment.	Host online events with limited space for sponsors to charge higher prices while networking the two core groups	Sponsors pay to be allowed in the online meeting and further to get a slot to present their products
Case C	Old	Provide place, food and service for a good time.	Create, cook and serve meals for customers in the restaurant	Get paid for every meal served
	New	Provide meals to eat at home.	Cook and deliver meals.	Get paid for every meal served
Case D	Old	Consult SMEs in strategic issues	Meetings, workshops and strategic development with customers	Charge for time used to consult the customer
	New	Sell hygiene products to business customers	Sell disinfectant to other businesses	Earn commission for every successful sale
Case E	Old	Produce and sell meat	Sell meat to restaurants only	Direct sales to business partners
	New	Produce and sell meat	Sell meat to customers and further retail organic products	Sales to customers through webpage, WhatsApp and on the farm

#### 4.1.1 Case A – distillery and beverage producer turns to disinfectants

Case A started to produce, bottle, and sell disinfectants as a business response to Covid‐19. This BM, although new to the industry (production of disinfectants is a state regulated BM in Austria and only specialized companies were allowed to produce them until the crisis started, which opened this BM to the industry), was not structural, and therefore can be seen as a focused BMI (modular and new industry). The variation to the existing BM is vast, requiring several processes to be altered to be able to implement the new BM. However, the core competencies in production and filling remained unchanged. With its value creation, the firm has changed from the production of alcoholic drinks to a disinfectant. The value proposition has also changed, as new customers are targeted with a different product; only the value capture element remained unchanged.

Although one of the firm’s employees developed the idea for the firm, it was decided not to make any changes when this person informed management. But management changed their mind a few days later, followed by the firm switching over to the production of disinfectant. The common goal made clear to everyone what they had to work on/change and how to implement the new BM. The firm’s flat hierarchy supported this process. As one of the employees responsible for the process said, ‘We all knew what we had to work on to get the product on the market. All departments were involved and you could see how motivated the employees were to implement this as quickly as possible.’ The implementation was supported through public authorities and partners who contributed their expertize to the processes. During the process, the firm was concerned that the existing brand and its high‐quality beverages would suffer from a negative image as a result of the change. But contrary to expectations, the interviewees noted that the firm’s rapid action has significantly increased awareness of its own strategic flexibility. It projected the notion that it can handle complex situations well, and is prepared for the future. Furthermore, it opened up new contacts and a new target group for the firm, which in the future will be reached via the current BM. So the experiences and new contacts obtained shortly after the introduction of the temporary BM led to positive effects on the core activities. Although the BM is currently generating good sales, there are no plans to pursue it long‐term.

#### 4.1.2 Case B – creating digital value for conference sponsors

This firm developed a specific online event as a response to the Covid‐19 crisis, addressing the needs of its customers and sponsors. This BMI can be described as evolutionary (modular and new to the firm), as no architectural changes were necessary and the BM of online events is used by others in the industry too. The variance to the existing BM is small, as most of the processes are still the same with the exception of room booking and catering. Within the scope of this temporary innovation, value creation has not changed, as it continues to focus on the networking of market participants. There were however changes in the value proposition. An event with personal character was brought into the digital space. The element of value capture has also changed: Through exclusive access to customers, sponsors are willing to pay more money per person reached. This firm approached a consulting firm with the concern that it would not be able to carry out their planned events. *‘We listened to the firm’s concerns that it would have to repay the money it had already received from the sponsors and developed a new concept based on the client’s skills and the characteristics of the event.’* The sponsoring contracts for the events had in most cases already been signed. During the crisis, it was noticed by all sides that affinity toward digital communication tools had increased significantly, which is why the firm decided to reproduce the conference within the framework of a digital meeting. A small group of the conference participants had up to this point been meeting on a regular basis for some time to exchange ideas over dinner. Over the course of the new BM, a handwritten invitation was sent out to participants who were to be networked together to create a similar personal environment over a drink. The participants are invited to digital conference rooms where the sponsors briefly present themselves. An opportunity that arose through the crisis was identified here, with extensive time invested to be able to hold the first run of the new event as quickly as possible. In the context of the crisis, innovation was of great importance, most notably in how it helped the firm avoid repayment to the sponsors. The interview partners pointed out that they can act more flexibly now, and see different new possibilities for creating revenue based on the new BM. A long‐term implementation is not planned at the moment, but was in fact discussed in light of the positive participant feedback received from the events.

#### 4.1.3 Case C – from pure cooking to selling toilet paper, masks, and delivery service

This firm responded to the crisis by starting a new delivery service, transforming into a retail store for consumer goods selling toilet paper and masks. The BMI can be described as adaptive (new to the firm and architectural) as it is new to the firm to sell goods, while others in the industry already established this before. Moreover, large structural changes were necessary which resulted in architectural changes. The introduction of a delivery service was well‐known in the industry, but required new processes and staff work profiles. The additional sale of consumer goods varies widely from the existing BM, creating further overall changes in the firm. And although the delivery service represented an extension of the firm’s value creation, the inclusion of consumer goods into the restaurant’s product portfolio was less common and altered its value creation. This additionally changed the value proposition from the processing of food to the sale of everyday consumer goods. By contrast, the value capture element hardly changed.

The idea for this came primarily from the owner, with the delivery service quickly implemented. This required internal processes to be changed, and the service staff trained accordingly (bring the food to the customers at their homes, not to a table in the restaurant). The BM for selling toilet paper was also implemented very quickly, as the firm was able to purchase it through existing supplier relationships within their network. Masks were designed and manufactured by a local tailor. In general, the financial impact of the new BM was minimal compared to the losses from the Covid‐19 lockdown. The delivery service only added minimal value, and has already been partly reduced. Although the sale of masks and toilet paper was profitable for the firm, there were reasonable concerns about the impact on the firm’s brand and reputation in the region. As a result, the margins on the products sold were low. The firm does not plan to continue this BM beyond the Covid‐19 crisis.

#### 4.1.4 Case D – a classic consulting firm gets into retail

This firm started distributing disinfectants via a European distribution network in response to the crisis. The BMI can be described as adaptive (architectural and new to the firm) as other consulting firms also work as sales companies, but for this firm major changes in their systems and approaches were necessary. Although the entry into trade is atypical for classical consulting firms, this firm has a long history in trade and numerous contacts. The owner stated how the decision was obvious for him, and the new BM is in line with his firm’s existing competencies and personal network. The new BM resulted in changes to all three elements, with the firm expanding value creation from advising customers to providing disinfectants. The sale of disinfectants represented a change in the value proposition. The firm creates revenue as a retailer now, and no longer just with consulting, changing the element of value capture as well. The idea emerged from the firm’s owner and through the closer network, with its joint cooperation starting shortly after the idea was born. The new BM could be implemented at this speed mainly due to a flat hierarchy and with the help of virtual organization with network partners who joined their core competencies, with the case firm taking over the distribution of the product. Creating sales strategies was a core competence that was already in place. After sales in Austria turned out to be profitable, the firm became a dealer to other countries as well, and the BM started to be perceived as a long‐term opportunity. Margins are currently low, with attempts being made to force other participants out of the market in the near future.

#### 4.1.5 Case E – an organic farmer sets out on new paths

This firm produces and sells meat to private clients from its farm, and operates a digital organic farm shop. Its BMI can be described as evolutionary (modular and new to the firm), as other farms follow the same system of a farm shop and only minor changes were necessary to change to the new customer segment. It is mainly based on the same core competencies. Only some infrastructure had to be adapted to sell the meat directly on the farm. This firm changed its value creation from a pure on‐site food seller to a digital shop. Furthermore, the live experience for the customers on the farm is an important factor for the firm; buying directly on the farm was still possible during the lockdown. While the value proposition is still the same (selling meat), the value capture of the firm has changed. Prior to the crisis, the farm was a B2B vendor and sold to restaurants only. However, this income stream vanished during the crisis, forcing the farm to come up with new ideas; its new BM was integrated quickly. In the near future, this firm plans to expand its digital shop with other organic regional products. As this firm has enjoyed a very strong brand image as an organic farm, there is a fear of damaging this image with the wrong products in the online shop. Furthermore, it hopes to not alter the existing relationship with business customers with the new BM. In general, with the BMI, the new sales pillar provides greater strategic flexibility to react to situations, and has also enabled new contacts. In the long run they aim to continue with the new BM, even though they expect that the original BM will be similarly important following the lockdown and the reopening of restaurants.

### 4.2 Cross‐case analysis

The following section concentrates on the cross‐case findings. We analyzed firms from different sectors and situations, with overall propositions for further research emerging upon closer inspection. Table 2 provides an overview of the case results. Our results indicate three important steps of a temporary BMI. These steps are the trigger and reason for a temporary BMI, insights into the integration of a TBMI and an outlook on the potential effects. Based on the detailed analysis of the cases we could develop a table that highlights similarities and differences among the cases and allows the identification of some patterns for temporary BMI. In particular our results of the cross‐case analysis focus on the three areas along the process of temporary BMI: (1) The trigger and reason for a temporary BMI. (2) The integration process and important issues. (3) The effects of temporary BMI. A fourth section deals with patterns we identified in our data that provide more details on temporary BMI.

**Table 2:** Cross‐case results and main insights

		Case A	Case B	Case C	Case D	Case E
Situation overview	Degree to which the crisis has affected^1^	Partly affected	Partly affected	Highly affected	Highly affected	Partly affected
Trigger and reasons for temporary BMI	Reason for integration^2^	Opportunity	Opportunity	Opportunity	Opportunity	Necessity
	Perspective of temporary BM	Short‐term	Long‐term possible	Short‐term	Long‐term	Long‐term
	Source of innovative idea	Employee	External consultant	Owner	Owner/network	Owner
	Relevance of temporary BMI^3^	Average	Average	Low	High	High
How and what changed	Network	Yes	Yes	No	Yes	Yes
	BMI typology	Focused	Evolutionary	Adaptive	Adaptive	Evolutionary
	Variance with existing BM^4^	Far	Near	Far	Near	Near
	Built on core competencies	Yes	Yes	Yes	Yes	Yes
	Branding issues	Yes	No	Yes	No	Yes
	Value creation	Changed	Same	Changed	Changed	Changed
	Value proposition	Changed	Changed	Changed	Changed	Same
	Value capture	Same	Changed	Same	Changed	Changed
Effects of temporary BM	Effects on Network	Yes	Yes	No	No	Yes
	Strategic flexibility	Yes	Yes	No	No	Yes

^1^ Self‐reported affectedness of the firm through the crisis (partly affected or highly affected).

^2^ Self‐reported reason why firms integrated into temporary BMI.

^3^ Self‐reported relevance of the temporary BM for the firm in regard to business performance.

^4^ Individually assessed by all researchers on the basis of the changes to the BMI elements.

#### 4.2.1 Finding 1 – trigger and reasons for temporary business models

The Covid‐19 crisis was the trigger for all of the firms to temporarily adjust their BMs, with all of them at least partially affected by the crisis, and Cases C and D highly affected. Only one case firm has already worked on its new BM prior to Covid‐19 (Case E). For the rest of the firms the change was based on newly emerging opportunities (Cases A, B, C, and D). ‘*Everyone started using online tools* [for meetings and communication]. *It was just more familiar to our target group and I decided this is an opportunity to do our networking activities online*’, so the consultant of case B. There are significant differences in the time perspective of the new BMs. While two firms (Cases A and C) only wanted to implement their new BM during the lockdown, there are two firms for whom the potential long‐term development was a decisive reason to adapt (Cases D and E). For firms seeking long‐term integration, the relevance of the new BM is high, while for others it is only average or low. The owner of case E explained this relevance to us: ‘*We have already started to change over shortly before the pandemic. Because of the pandemic, our main source of sales collapsed. Restaurants had to close. We therefore had to work even faster. That was incredibly important because we couldn't sell anymore. But that is also how it will be in the future, that we will sell directly to private individuals*.’ Moreover, the companies that integrated temporary BMI with the intention to sustain it over the long run mentioned that they also invested a significant amount of resources. An important basis for the introduction of a temporary BM is the initial idea and observed opportunity. This may come from very different sources. In case A, an employee had the idea, in case B an external management consultant, and in cases C, D and E the owner. The CEO in case A stated: ‘*Our employee realized that the competitors in more seriously affected regions already started to work on this opportunity. He convinced us to also do this*.’ The statement shows the flat hierarchies and short command lines in SMEs and is an indication that in SMEs innovation can also be initiated quickly by employees and external parties in order to master a crisis.

#### 4.2.2 Finding 2 – integration process, branding and business model elements

Most of the temporary BMs observed emerged from the crisis (Cases A, B, C, and D). For these firms, the rapid exploitation of their respective opportunity was of great importance. As a result of the crisis, capacities were freed up in the firms to take advantage of these opportunities. The CEO of case D explained: *‘In the course of the crisis, some major projects were cancelled or postponed. This gave them enough time to implement this business model quickly’* (Case D). Rapid implementation was achieved with the help of free capacities^2^ (Cases A and C), by external consultants (Case B), and by using the existing network (Cases C and D). Case E is the only one that carried out the implementation itself. One reason for the rapid implementation of the temporary BMs was that they were based on existing core competencies at all of the firms. The case firms created BMs around new opportunities but in line with their established competence base. The companies also described how they used their core competencies: Case A who primarily produces spirits could not sell the same amount as restaurants were closed. They explained that they could build on their existing processes and competences of following a receipt and producing and mixing different ingredients, when they started to produce disinfectant rather than spirits. Also in packaging and distribution they could build on existing capabilities. The CEO mentioned: ‘*We don't do anything different today than we did in the past. We follow a recipe and mix the necessary ingredients together to make a product.’* Case B, who had offline events with sponsors, for example still sees its competence in matching the right people together. Instead of doing this offline they do it online now. As firms relied on these existing competences, residual required competences that were not readily available were very few and could be built up quickly via the existing network in order to be able to react quickly (Cases A, B, D and E). This made the implementation of the temporary BMI very fast and efficient. We observed a considerable variance (Cases A and C) between existing and temporary BMs for those firms who only see their BMI as a short‐term opportunity. On the other hand, firms who indicate that they are planning or are already working on a long‐term continuation of their temporary BMI (Cases B, D, and E) obviously conduct changes in their BM that are generally more incremental in nature. Still, in general, even in the cases where more radical changes were carried out, the existing core competencies formed the decisive basis for establishing these BMs (Cases A and C). For three firms (Cases A, C, and E), a good reputation and a strong brand belong to these core competencies. These firms were incidentally concerned about the effects of the temporary BM on their brands (Case A, the most affected firm), thus ensuring that the temporary BMI is aligned with the existing brand reputation. An employee explained: *‘We produce high quality spirits and are now a manufacturer of disinfectants. We have to be incredibly careful to draw a clear line here in communication.’* An analysis of the media, the homepage and their social media accounts interestingly showed that these case firms even prevented to inform the public about their newly integrated BM. Firms that have established a significant, well‐known brand among private customers in their daily business are more concerned about their reputation than purely B2B firms. From this perspective, the organic farmer who has to pay attention to the organic origin of the products sold in his local and digital farm shop had to consider branding in order not to lose the trust of his customers. Secondary data show that they put a lot of emphasis into communicating their organic status throughout their means of communication. The restaurant, which enjoys a solid reputation throughout its region, also had to keep this in mind. All these firms had to design their new BM in a way that did not damage the existing brand.

Our results indicate several changes in the BMI elements and different BMI typologies. First we see that at least two of the three elements changed in all of the cases. In one case (Case D), all three elements changed. A special situation could be analyzed for the value capture element, which is not changed if companies’ temporary BM is far from the existing one. This could be due to the fact that these firms do not plan to integrate the temporary BM into the firm in the long‐term, i.e., these changes will take place while continuing to focus on existing BMs and changes in value capture are more complex than in other dimensions. Second, we could find that based on the *BMI Typology* focused (new to the industry and modular), evolutionary (new to the firm and modular) and adaptive (new to the firm and architectural) BMI was used to establish a new BM. Two companies (Cases C and D) followed an adaptive BMI another two (Cases B and E) did an evolutionary BMI while only one firm integrated a focused BMI (Case A). The results indicate a relationship among adaptive BMI and the affectedness of the existing BMI, which is further described in finding 4.

#### 4.2.3 Finding 3 – the effects of temporary business models

The results showed that the introduction of a temporary BM had positive effects on the case companies. Cases A, B, and E noted positive effects on their network. They mentioned that the heterogeneity increased through new contacts which will help in different situations, moreover they highlighted the positive effects of contacts in various industries that think about solutions differently. Finally they mentioned that their total customer base increased as they addressed new customer segments, which may allow them to address these with their original BM in the future. In addition to our expectations, three cases (Cases A, B, and E) described BMI creates beneficial effects on strategic flexibility, which resulted from a deeper reflection and understanding upon their own core competencies and the potential strategic opportunities that could be addressed through these. The companies realized that on this basis the dimensions of a BM can be successfully adapted to changing conditions. The CEO of case A explained their increased strategic flexibility in the following: *‘We have seen what we are capable of when we enter a difficult situation. Our team has shown us that we can also achieve sales in other areas when a change is needed.’* The managing partner of case B further explained: ‘*It was good to see that we are flexible enough to adapt to this crisis. Our competitors just cancelled their events while we could still generate revenue*.’ Moreover, the new BMs allowed these firms to be more flexible through the generation of additional revenue streams, helping to better adjust to changed situations while working on new value creation.

#### 4.2.4 Finding 4 – temporary business model patterns

Despite the limited number of cases the results indicate two general patterns in temporary BMI as a response to crisis. The first pattern differentiates primarily those companies that are significantly affected from those that were less negatively affected by the crisis. This level of affectedness leads to different results and directions. The crisis did not have the same effects on all the companies. The ones that were affected to a greater degree (Case C and D) both engaged in adaptive BMI, which means that their innovation was new to the firm and architectural. As these companies were more constrained by the crisis, they could no longer follow the existing BM. Due to the severity of restrictions they had to do *architectural* changes in their BMs to generate new revenues. This huge change of the existing BM created a situation in which none of the effects we realized with other companies (positive effect on network and positive effect on strategic flexibility) could have been discovered. On the other side companies that were less negatively affected changed their BMI more incrementally via modular changes and could consistently benefit from the positive effects of the new BM on strategic flexibility, on the existing network and on the existing BM. They reported that they are more aware of their core competences and could easier respond to environmental changes based on this experience in the future which allows them to be more flexible from a strategic perspective. On the other side they mentioned that they plan to leverage the new contacts they reached with their BMI to do cross sales and develop innovative solutions for this new segment. Moreover, they mentioned that the experience during the creation of the temporary BM helped them to further develop the old BM.

The second pattern differentiates the perspective companies have on the new BMs. Companies who planned a long‐term continuation of their temporary BMI show a different pattern of behavior as compared to those companies with a rather short‐term perspective. It is realizable that the strategic planning horizon behind a temporary BM does have effects on the investment companies are willing to do, the distance to the existing BM, branding and also the value capture dimension. Companies that change their BM just for short‐term survival are fine with a larger distance between the existing BM and the new BM. As they do not plan to implement it in the long‐run they are more concerned about their brand and that the temporary BMI could have negative influence on it. Moreover, our data indicate that companies that only follow a short‐term implementation try to keep going with the same *value capture* as before. On the other side, companies that plan their new BMs for a long‐term perspective actively mention that they invested money to integrate the new BM. For example, an onsite visit at Case E showed that they invested into new infrastructure to build cooling capacity and a store on their farm.

## 5 Discussion and conclusion

### 5.1 Synthesis of key results

**Figure 1:**
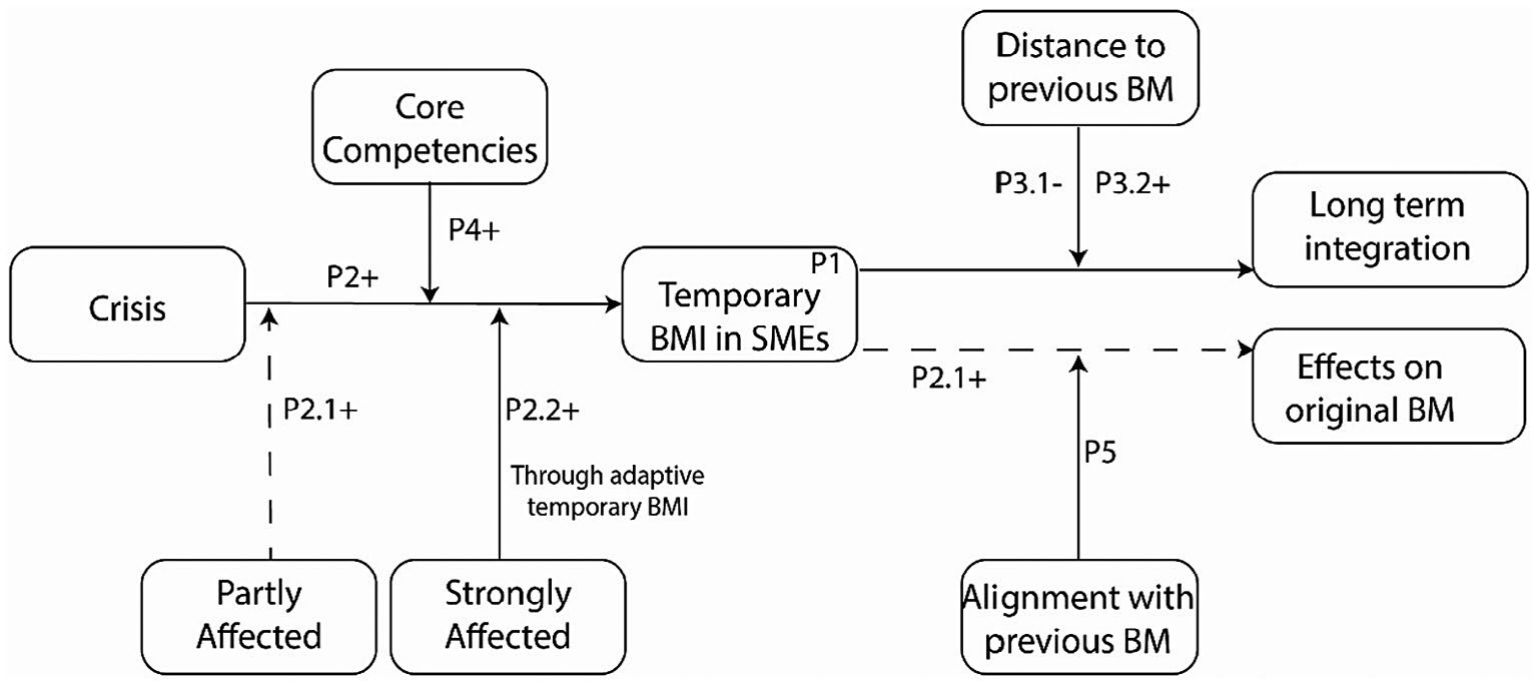
Overview on the propositions of temporary BMI.

In the following section we synthesize our results and create five core propositions on temporary BMI. They explain the relationships of our core constructs and should encourage future research on this topic based on our empirical temporary BM foundation. At the end of this section we provide an overview on these propositions (Figure 1).

This study focuses on SMEs and indicates that these companies use temporary BMI to survive a crisis. SMEs are characterized by various criteria. These include the liability of smallness, which means that SMEs have fewer resources available in times of crisis (Eggers, 2020). Due to the limited resource availability, certain crisis management strategies explained through Wenzel et al. (2020) could at maximum be used for a short period of time. A pure retrenchment or persevering strategy cannot be pursued over a long time, without new revenue streams as a SME. Authors further explain that SMEs have to be innovative to survive turbulent environments (Le Nguyen and Kock, 2011), which is clearly indicated through our analysis. Thus, in particular for SMEs a proactive innovation strategy based on temporary BMI seems to be a feasible solution to manage an exogenous crisis. On the other side SMEs have lean structures which allow them to be more innovative. As our data show it is possible for employees and external stakeholders to persuade the management in SMEs to pursue new ideas. Furthermore, the management is more directly involved in the firms’ operations, leading to direct and quick opportunity recognition. Concluding SMEs are more likely to engage in temporary BMI as other strategies are not suitable for a long period and the lean structures allow them to easier implement new ideas.

Proposition 1 Temporary BMI is particularly appropriate for SMEs during crisis.

Our cases clearly show that an exogenous crisis is a trigger for temporary BMI. In all investigated cases the crisis triggered the innovation process. This is in line with existing literature describing that crises lead to new innovation opportunities (Brockner and James, 2008). Saebi et al. (2017) explained that firms tend to adapt their BMs when they are under threat. In the case of an exogenous crisis, these opportunities may be temporary. Temporary BMI differs in companies that are severely or only partly affected by the crisis. Significantly constrained companies may be in a need to move further away from the actual BM in order to be able to create new sources of revenue. These companies must do an adaptive BMI based on Foss and Saebi (2017) which forces the firm into architectural changes of the existing system. Therefore, the changes in these companies are more severely and they are not able to profit from positive effects on their network (new contacts in different industries to learn from or customers they can also address with the original BM) or strategic flexibility. Less constrained companies benefit from stronger positive effects of the new BM on the existing BM through the newly gained experience and feedback outside their home industry, the network and their strategic flexibility. While other authors investigated strategic flexibility as an antecedent of BMI (Bock et al., 2012; Clauss et al., 2021). Schneider and Spieth (2014) experimentally showed that BMI indeed increases the strategic flexibility of firms across different dimensions. In line with this, our study showed that those firms facilitating temporary BMI could increase their strategic flexibility and may therefore develop a capability to better react to potential future crises (Muhic and Bengtsson, 2021). SMEs’ active responses create experiences in coping with complex situations, and the foundation for further BMIs as a result (this was explained by Zook and Allen (2003) in the context of multinationals).

Proposition 2 An exogenous crisis serves as an initiator of temporary BMIs in SMEs.

Proposition 2.1 If an exogenous crisis strongly affects an existing BM this leads to the implementation of a temporary BMI through adaptive BMI.

Proposition 2.2 Under circumstances where an exogenous crisis affects an existing BM only partly a temporary BMI has positive effects on the existing BM, strategic flexibility and increased business network.

While Kraus et al. (2020a) describe temporary BM adjustment as a short‐term response to crisis, some of our cases clearly indicated that these companies intend to transform temporary BMs into long‐term BM changes. For these cases their temporary BM is not only an opportunity that works during the crisis but can also create revenues after it. In particular those companies, whose temporary BMI is close to the existing BM, are actively planning such a long‐term integration of their temporary BMI. If a BMI is only incremental and creates new revenue streams, it is a case for long‐term implementation. It is important to see future potential for the temporary BM after the crisis too and it should be compatible with the existing BM to prevent harming the core BM. In this situation it makes sense to integrate the new BM also in the long run, especially if it is possible to integrate it into the existing strategy.

On the other side we observed companies that do not plan a long‐term integration of their temporary BMI. Interestingly, while these companies follow BMs that are more fundamentally different from their existing one they still do not change their value capture dimension. The larger distance to the existing BM allows them to do a short‐term change to create new revenue streams but it forces them to get back to the old one if they would like to go with the existing strategy (Casadesus‐Masanell and Ricart, 2010). Changes in the value capture dimension are more complex than the other dimensions and were shown to require system wide adaptions that could not be achieved through temporary BMI (Clauss et al., 2021). This may explains why the case companies did not change their value capture for temporary BMI even if more radical BMI were conducted.

Proposition 3.1 Temporary BMI that are closer to the traditional BM of SMEs show potential for long‐term integration and may thus be a basis for long‐term BMI.

Proposition 3.2 Temporary BMI that are far to the traditional BM of SMEs are only implemented for a short‐term perspective.

Our results indicate that the quick response through temporary BMI in SMEs is made possible as the case companies rely on existing competencies and slack resources as a basis for their temporary BMIs. Moreover, leveraging existing personal and business contacts increases the speed of BM implementation, especially during a time where free capacities are available due to restricted existing BMs. This demonstrates that temporary BMI may be facilitated through a firms core competency base. The literature examined how core competencies are an essential factor for general BMI (Matzler et al., 2013). Moreover, it is suggested that incremental BMI should be developed in line with core competencies (Prahalad and Hamel, 1990). Zook and Allen (2003) further explained that it is important to keep situations of change manageable by building on existing capabilities and working one step at a time. Changing environments lead to an increasing complexity, which can be handled using limited changes in the firm that build on existing competencies. Leveraging the network and core competencies helps to implement a BMI in SMEs.

Proposition 4 After an exogenous crisis core competencies leveraging is used for temporary BMI in SMEs.

In line with this argument our findings further suggest that firms may implement temporary BM quickly, albeit with foresight. This is illustrated by the obvious concern of our case companies that temporary BMI my harm their brand reputation and therefore their existing BM. Especially, companies that only create a new BM for the short run have a huge interest to protect their reputation and core BM. The reputation of our case companies is often described as a core competence. They are well known in their industry and respected for good quality. Therefore, the creation of short‐term revenue streams through a temporary BMI must be possible without risking this reputation. This effect is also visible in other areas. In their study, Zook and Allen (2003) showed that growing firms are always careful not to weaken their existing core, and basically only pursue one opportunity after another. This sequential approach results in easier processes and allows a BMI to be implemented without harming the core of the firm, keeping the possibility to go back to the core BM alive. Companies that plan to integrate their temporary BM in the long run too are less afraid of harming their reputation. As proposition 3.1 and 3.2 already explained these are also the companies where the existing BM is not that far from the new one. By pursuing an incremental change of the BM the possibility to harm the old one is more limited. On the other side companies that are afraid their temporary BMI could harm their reputation are engaged in more radical changes of their BM.

Proposition 5 Temporary BMIs should be done without harming the existing BM.

In total we proposed five core propositions with individual sub‐propositions that could better describe temporary BMI. Figure 1 summarizes all these propositions into a model to show their overall connections and provide a better understanding of the investigated variables.

### 5.2 Contribution to research

Our study makes two important contributions to the literature. First, we contribute to the literature on crisis management in SMEs by identifying and elaborating a management mechanism that allows firms to survive and may even improve during a severe exogenous crisis such as the one caused by the Covid‐19 pandemic. A quick and successful response to crises is an important issue for SMEs due to their limited size and lack of resources (Eggers, 2020). Our findings are in line with previous studies showing that exploration strategies, engagement in innovation, and entrepreneurial orientation may help firms to survive a crisis (Eggers and Kraus, 2011; Vargo and Seville, 2011). Beliaeva et al. (2020) relate the positive effect of an entrepreneurial orientation during a crisis to firms’ ability to identify opportunities during crises. We substantially add to this previous knowledge as we further develop initial arguments on short‐term BM adaptions (Kraus et al., 2020a) and further explain the concept of temporary BMI as response strategy for SMEs to create new revenue streams and increase liquidity in cases were the existing BM is negatively affected by a crisis. Specifically, more incremental adaptations of existing BMs provide opportunities for SMEs to test alternative BMs which can be pursued during/following a crisis. In addition to that, our research shows that going through a temporary BMI increases the strategic flexibility of firms, supporting previous research of Ates and Bititci (2011) who show that current change processes also increase SMEs resilience for future crises.

Second, we contribute to the BMI literature by substantiating temporary BMI as a new form of BMI. Kraus et al. (2020a) created the foundation for temporary BMI as a short‐term response to crisis. While BMI as a response to changing external conditions has been presented as a costly and time‐consuming change that is radical by nature (Snihur et al., 2018; Clauss et al., 2021), temporary BMIs create the possibility to more incrementally change the BM in a short period of time and with limited resources. This is possible as temporary BMI relies on exiting core competencies of the firm and thus – although the dimensions of the BM are altered – can reduce the challenge of a BMI (Prahalad and Hamel, 1990). Therefore, our research brings a new perspective to the emergence and rigidity of BMs. Casadesus‐Masanell and Ricart (2010) initially explained that BMs are the result of a firms long‐term strategy. In this view, these rather rigid structures could thus not be altered at a short‐term basis and reactions to changing environmental conditions could only be conducted through minor tactical maneuvering in the scope of residual BM flexibility. In contrast to this, we find that indeed, temporary BMI is possible under certain conditions. This may be explained based on Mintzbergs (1978) idea of emergent strategies. Under extreme conditions such as a crisis caused by a pandemic where the underlying assumptions of the deliberate strategy are not valid anymore firms may develop these emergent strategies, which will then be operationalized through a temporary BMI. We thereby show that despite previous assumptions, temporary changes of BMs are possible independent of the deliberate long‐term strategy of the firm.

However, our results clearly demonstrate that these temporary BMI are established based upon the existing core competencies. Thus, although temporary emergent strategies may lead to temporary BMI under situations where existing deliberate strategies are rendered obsolete, these are still in line with the resource based foundations of the original strategy leading to BMIs that are less radical and address adjacent industries. In general this finding is in line with ideas of the resource based view (Barney, 1991), advocating strategy definition based on unique firm resources. Companies that are aware of their own core competencies have the opportunity to scale in adjacent domains through an adaption to a changed environment based on emergent strategy and a temporary BMI. This behavior creates new revenue streams in areas that are not or less affected by the crisis and create a better chance of survival.

We show that temporary BMI is a successful crisis management approach during exogenous crises when the core BM is severely affected by changing external conditions. However, our finding may also be more generalizable to BMI under other conditions. Arguably, temporary BMI could also be a relevant approach during normal times to help innovate BMs and test alternative ones. Through, such temporary BMI, new BMs can be tested, changed, and improved. In line with previous research (Sosna et al., 2010; Andries et al., 2013), temporary BMI constitutes a resource efficient opportunity to experiment with new BMs without putting the core BM at risk. This experiment can lead to a long‐term integration of the new BM or to a change back to the existing one. A key to pursuing an experimental approach based upon temporary BMI is not to endanger the existing BM. We add to this discourse by showing that one important criterion to enable this process in incumbent firms is that the BMI relies on firms’ existing core competencies and is connected to the core business (Zook and Allen, 2003). If the BMI is setup to be temporary and draws on the core competencies of the organization, a return to the core BM is always possible, resulting in limited risks for the firm. This option to return to the core BM is further enabled through a separation of the old and the new BM in time. Temporarily shifting to a different BM or running two BMs simultaneously is facilitated if the new temporary BM does not harm the old one (Markides, 2013). Moreover, the proximity of a BMI to the core competencies of the firm allows it to be implemented quickly. A core BM does not need to be forcibly reduced to implement a temporary BM, which could perhaps promote parallel synergetic BMs. In this vein, temporary BMI may be a potential approach to implement and manage multiple BMs (Winterhalter et al., 2016). As suggested by Markides (2013) a temporal separation may be a solution to manage two competing yet integrated BMs. This may be facilitated through temporary BMI. Furthermore, the initial findings of this study indicate that in the long run existing BMs can also benefit from temporary BMIs through an extension of the network into new industries and an improved knowledge of the own core competencies.

### 5.3 Managerial implications

This study shows that a sudden and unexpected crisis can also unlock enormous potential for firms, provided they are open‐minded, willing, and prepared to search for potential opportunities during a crisis. Firms can recognize new opportunities in a rapidly‐changing environment, and must be aware of their core competencies to recognize opportunities that might be far removed from existing BMs. By exploiting their own network, and applying their own competencies, unused resources can be quickly bundled into new BMs during a crisis. With a global crisis, these BMs can serve not only a purely economic purpose, but a social one as well. Rapid responsible innovation is an ‘innovation developed in a short period of time in a state of emergency with the hope of protecting people and saving lives’ (Gutierrez‐Gutierrez et al., 2020). Some of the firms examined in the course of this study can be associated with this approach, and serve as examples of how to sustainably operate long‐term.

In addition to the issue of sustainability, our study shows clear positive effects for firms. The introduction of a temporary BM leads to positive effects on the current BM. The understanding of the own BM changes by looking at other industries and processes, and gaining new customer groups. Above all, rapid reaction by firms leads to increased flexibility and reveals a firm’s potential. A sharpened view of the own core competencies can have long‐term positive effects.

### 5.4 Limitations and outlook

This study was dedicated to the analysis of temporary BMs, providing first insights into the effects they can have and how they can be implemented. Numerous other propositions have arisen as a result. This study is limited by its scientific approach. Only firms experiencing BMI during a crisis were specifically examined and all these firms have been SMEs. The companies came from low tech industries, which were more affected by the crisis. The impact of the Covid‐19 crisis on other firms was not addressed. However, it is to be expected that the crisis will also lead to significant changes at other firms. Nevertheless, some firms profited during the crisis and we cannot draw any conclusion on these companies. To overcome these issues the relationships identified in this research should be investigated in a quantitative analysis. Furthermore, due to the current timing, it is not yet possible to conclusively say what effect the introduction of a temporary BM will have on the overall performance of a firm, or any resurgences following a crisis. The long‐term effects of temporary BMI on existing BMs as well as on firm performance will have to be the objective of future research. Lastly, we focused on temporary BMI triggered by an exogenous crisis that forced firms to develop a new BM in order to ensure the survival of their firm. However, this study did not investigate other more proactive antecedences of BMI as discussed in the literature, such as strategic agility (Clauss et al., 2020), learning (Berends et al., 2016) or knowledge management (Hock‐Doepgen et al., 2021). Future research might therefore investigate the antecedences of temporary BMI besides or in addition to the external shock caused by the crisis. Moreover, the results in this study are only a first analysis of temporary BMI. They are only one possible way to pursue these kind of BMs. Future studies should refine this model and show different approaches based on other cases or quantitative research.

## Supporting information

Supplementary Material

## Notes

^1^
Henderson and Clark (1990) defined architectural and modular innovation. This definition must not be confused with the BMI Typology of Foss and Saebi (2017), that also uses the terminology architectural and modular. Their terminology is built on fifteen years of BMI research and describes the scope of a BMI.

^2^
As a result of the Covid‐19 lockdown, new capacities for strategic considerations and new operational implementations were freed up in the course of operational cutbacks. We describe these as ‘free capacities.’
