# Supplementary material for: Temporary business model innovation – SMEs’ innovation response to the Covid‐19 crisis
Source: R&D Management. 2021 Aug 26;52(2):294–312. doi: 10.1111/radm.12498 (PMC8662028; doi:10.1111/radm.12498)
Supplement: Supplementary file 1 — Supplementary Material [file RADM-52-294-s001.docx]

**Temporary business model adjustment – SMEs innovation response to COVID-19 crisis**

# ***Case description***

This study is based on five heterogeneous cases to gain an insight into the statements of SMEs from different industries and origins regarding their temporary business models. The firms were promised anonymity in the course of the data collection. Therefore, they will be described in the following as Case A, B, C, D, and E. At this point, a short description of the firms is given to be able to classify them. Table 1 provides information about the SMEs, their main business models, as well as their polar type (i.e. the degree to which the crisis has affected the SME).

| **Case** | **Country** | **Current business model** | **Data Sources** | **Degree to which the crisis has affected** | **Temporary business model adjustment** |
| --- | --- | --- | --- | --- | --- |
| Case A | AUT | producer of spirits | 2x Interview Analysis of homepage Internal reports Social media analysis Media analysis | partly affected | Producer of disinfectant |
| Case B | FL | Software as a Service and Events | 3x Interview Internal Reports  E-Mails Observation of event | partly affected | Online networking |
| Case C | GER | Classic restaurant | 2x Interview Social media analysis Media analysis | highly affected | Mask and toilet paper seller Delivery of food |
| Case D | FL | Business Consultant | 1x Interview Homepage Social media analysis Internal reports | highly affected | Seller of disinfectant |
| Case E | AUT | Producer of food and sale to businesses | 2x Interview Homepage Social media Onsite visit on farm | partly affected | Seller of food to customers |

Table 1: Overview of the cases and their specifications

## *Case A*

*Case A* refers to a firm that specializes in the production of schnapps products. The firm has more than 200 employees and was founded in 1886 and is a family firm. Besides the distillery of highly potent alcohol, the firm also produces juices and other beverages. The main season of the firm is in winter, with the most important customers being ski huts and après-ski bars. In recent years, the firm has achieved strong growth figures and, above all, has expanded into further countries. It´s main business model is based on the sale of alcohol and beverages to retailers and restaurants. However, a B2C model is also part of the strategy. So they sell directly to end consumers via an online shop and a shop near the production facility. The B2B pillar of the firm is the more important one and creates most of the revenue. With the start of the crisis sales to business customers suffered temporarily and the winter season ended early. However, the firm's online shop received more orders during the crisis. Private customers in particular purchased more products. So, during the crisis the much more important B2B sales decreased extensively, while the B2C sales increased at least at the beginning of the crisis and got back to normal.

## *Case B*

This firm works as an event planer and consultancy firm in the finance industry. The firm was founded in 2009 and employs eleven people. The firm holds an annual event, which has developed into a cash cow. The focus of the event is on the networking of market players and takes place on a smaller scale. At the first day only the sponsors (asset managers) and the exclusively selected asset owners meet for dinner. At this stage most of the networking is done and they get to know each other. On the second day there is a fullday event witch sponsors and asset owners in different discussion rounds. After this fullday event the sponsors and asset owners meet again for dinner. Every event only allows 3 to 5 individual sponsors. A sponsoring contract therefore is very exclusive. Most of the value is created through the inofficial dinners the day before and at the day of the event itself, where the other participants are not invited. However, a continuation of border controls and a ban on events has meant that the network meeting, which has become a cash cow, cannot take place.

This company further engages in another business model which was not affected by the crisis and therefore already works on multiple business models.

## *Case C*

*Case C* is a firm from the hospitality industry. The restaurant offers its customers in Germany reasonably priced evening meals and regionally known lunch menus. In total, the SME usually employs about 45 people, although due to the COVID-19 crisis, in addition to short-time work, individual terminations were necessary. There are 180 seats in the restaurant and another 170 seats in the garden. The restaurant is operated classically with staff in the service and in the kitchen. It also operates a counter sales service, where meals can be bought for take away. In the course of the COVID-19 crisis, the firm has been particularly hard hit. Due to the non-pharmaceutical measures, the firm had to discontinue its restaurant operations except for the counter sales, where national restrictions could be fulfilled. The firm does not have any experience with food delivery and did not have any special equiptment for this business model.

## *Case D*

*Case D* is based on a classic consultant firm in Liechtenstein. The firm is 10 years old and is based on a partnership agreement. There are four people in the company today. The consulting firm has always been distinguished by its ability to seize opportunities and offer consulting services in a wide range of industries. In addition to classic consulting, the focus is on accompanying firms over the long-term and actively participating in the operative implementation. The consulting firm was created from a sales consulting activity. Even today, many orders still deal with the conception of a sales model. The COVID-19 crisis has led to the fact that numerous customers with their projects planned for spring 2020 have cancelled or postponed them indefinitely. As a result, the firm has lost planned sales and existing capacities have been released. Based on the existing competence of sales the firm startet to work on a new BM in the sales section and decided not to pursue it for other clients but for itself.

## *Case E*

*Case E* deals with one of the largest organic farmers in the region and employs a total of five employees. The firm sells beef and milk. It also processes and sells wood and other by-products of livestock farming. While they only sell their beef to restaurants, the milk is sold to a regional dairy.

The family owned firm is managed by the youngest generation. The manager is only 25 years old and took over the farm in 2018. The former manager is still in the firm and supports in strategic decisions and operative work. The firm is not subject to seasonality, however, animals that are ready for slaughter must be slaughtered and sold immediately. This means that the company cannot react spontaneously to a slump in order intakes, as has happened during the crisis. The lockdown for restaurants, their primary customers, created a complete drop in B2B demand and the firm had to search for other ways to sell their products.

While the B2B market for meat completely collapsed due to the lockdown, the company, as an organic farmer, could continue to sell the milk it produced. However, since the sale of meat accounts for the bulk of the generated revenue, an alternative solution had to be found.

# ***Interview Guide***

**Assessment of the situation**

To what extent is your company affected by the Corona crisis and how do you perceive this situation?

What acute measures did you take after the onset of the crisis?

What longer-term changes are you thinking about so that your company not only survives the corona crisis, but ideally emerges from the crisis stronger?

**Focus on the Temporary Business Model Innovation**

Who had the idea for the temporary business model and what does it look like?

What is the significance of the business model and how obvious was the idea?

Why did you decide to introduce a temporary business model?

How was the new business model implemented in such a short time?

What influence does the new business model have on existing processes and relationships?

Is there anything else you would like to mention?
